# YOLOv8-MU: An Improved YOLOv8 Underwater Detector Based on a Large Kernel Block and a Multi-Branch Reparameterization Module

**DOI:** 10.3390/s24092905

**Published:** 2024-05-01

**Authors:** Xing Jiang, Xiting Zhuang, Jisheng Chen, Jian Zhang, Yiwen Zhang

**Affiliations:** School of Tropical Agriculture and Forestry (School of Agricultural and Rural, School of Rural Revitalization), Hainan University, Danzhou 571737, China; xingjiang@hainanu.edu.cn (X.J.); zhuangxiting@hainanu.edu.cn (X.Z.); jishengchen@hainanu.edu.cn (J.C.); 20213005613@hainanu.edu.cn (Y.Z.)

**Keywords:** object detection, deep learning, YOLOv8, UniRepLKNet, Swin transformer, SPPFCSPC

## Abstract

Underwater visual detection technology is crucial for marine exploration and monitoring. Given the growing demand for accurate underwater target recognition, this study introduces an innovative architecture, YOLOv8-MU, which significantly enhances the detection accuracy. This model incorporates the large kernel block (LarK block) from UniRepLKNet to optimize the backbone network, achieving a broader receptive field without increasing the model’s depth. Additionally, the integration of C2fSTR, which combines the Swin transformer with the C2f module, and the SPPFCSPC_EMA module, which blends Cross-Stage Partial Fast Spatial Pyramid Pooling (SPPFCSPC) with attention mechanisms, notably improves the detection accuracy and robustness for various biological targets. A fusion block from DAMO-YOLO further enhances the multi-scale feature extraction capabilities in the model’s neck. Moreover, the adoption of the MPDIoU loss function, designed around the vertex distance, effectively addresses the challenges of localization accuracy and boundary clarity in underwater organism detection. The experimental results on the URPC2019 dataset indicate that YOLOv8-MU achieves an mAP@0.5 of 78.4%, showing an improvement of 4.0% over the original YOLOv8 model. Additionally, on the URPC2020 dataset, it achieves 80.9%, and, on the Aquarium dataset, it reaches 75.5%, surpassing other models, including YOLOv5 and YOLOv8n, thus confirming the wide applicability and generalization capabilities of our proposed improved model architecture. Furthermore, an evaluation on the improved URPC2019 dataset demonstrates leading performance (SOTA), with an mAP@0.5 of 88.1%, further verifying its superiority on this dataset. These results highlight the model’s broad applicability and generalization capabilities across various underwater datasets.

## 1. Introduction

In the sustainable management of marine resources, the accurate detection and localization of underwater resources are crucial. Remotely operated vehicles (ROVs) and autonomous underwater vehicles (AUVs) play an irreplaceable role in locating marine life, mapping the seabed, and other underwater tasks. The scope of these applications extends from monitoring marine species [1] to underwater archaeology [2] and aquaculture [3]. However, designing a fully functional AUV requires the integration of advanced technologies such as target detection [4,5], tracking [5,6,7,8], grasping [9], human–machine interaction [10], autonomous control [8], and multimodal sensor integration [11]. ROVs and AUVs play a central role in the development of underwater target detection technology. They assist in mapping the seabed and locating potential obstacles by identifying the terrain and biological categories of the seabed, and they are also used to inspect underwater facilities. Although existing target detection technologies perform well in extracting low-level features such as shapes, outlines, and textures [12,13,14], the recognition of these features often lacks precision and is slow in complex underwater environments.

Underwater target detection faces numerous challenges. Firstly, the absorption and scattering of light by water cause unstable lighting conditions, significantly reducing the contrast between targets and their backgrounds [15]. Secondly, factors such as water currents, suspended particles, and foam can cause image blurring and distortion, thereby reducing the recognition accuracy [16]. Moreover, the diversity of the targets in underwater environments, with significant differences in appearance, size, and shape among marine organisms, adds complexity to detection tasks [17]. Finally, various noise and disturbances such as water waves, bubbles, and floating debris further interfere with the detection and identification processes [18]. To address these challenges, researchers have proposed several improvement strategies, including expanding the receptive field, enhancing the feature expression capabilities, implementing multi-scale information fusion, and facilitating comprehensive information interaction [19,20,21,22,23,24,25,26,27,28,29].

Firstly, increasing the receptive field helps to better capture the contextual information and environmental characteristics of targets, which is crucial in making accurate predictions in complex underwater environments. Deep convolutional neural networks (CNNs) have demonstrated their exceptional processing capabilities across various domains in recent years. Numerous studies have shown that by adjusting the depth of the CNN and the size of the convolutional kernels, the network’s receptive field can be effectively expanded [19]. These strategies are particularly beneficial in tasks requiring dense predictions, such as semantic image segmentation [20,21], stereo vision [22], and optical flow estimation [23]. Ensuring that each output pixel is influenced by an adequate receptive field enhances the accuracy and robustness of the algorithm.

Additionally, the adoption of nonlinear activation functions, the integration of attention mechanisms, and the application of data augmentation techniques [24] can significantly enhance the network’s ability to process the input data, thereby improving the accuracy in recognition, classification, or localization tasks. Techniques such as feature pyramid networks [25], multi-scale fusion modules [19], and Atrous Spatial Pyramid Pooling (ASPP) [26] enable the generation of feature maps at various resolutions, effectively integrating feature information from different scales to enhance the system’s recognition capabilities. Advanced architectures such as standard Transformers and their variants [27,28] and DenseNet [29] further boost the model’s performance and adaptability by managing complex data structures.

In summary, in the field of underwater target detection, existing research has been conducted on the aforementioned improvement strategies. However, there are still significant shortcomings in considering the complex underwater environment comprehensively and achieving higher precision. To address this, this paper introduces the improved YOLOv8-MU model, which integrates advanced computer vision technologies such as large kernel blocks (LarK blocks) [30], C2fSTR, and Spatial Pyramid Pooling Fully Connected Spatial Pyramid Convolution (SPPFCSPC) [31] with attention mechanisms to enhance the model’s receptive field, multi-scale fusion capabilities, and feature expression abilities. Furthermore, by incorporating a fusion block [32], we have further enhanced the model’s performance in multi-scale feature fusion, optimizing the feature aggregation process and thus improving the flow of gradient information and network performance at various levels. Additionally, the model has been optimized to accommodate resource-limited edge devices, with an improved loss function (MPDIOU) [33] that enhances the precision of localization for targets with unclear boundaries.

The experimental results on the URPC2019 dataset demonstrate that the YOLOv8-MU model achieved an mAP@0.5 of 78.4%, which represents a 4.0% improvement over the original YOLOv8 model. Additionally, the model reached an mAP@0.5 of 80.9% on the URPC2020 dataset and 75.5% on the Aquarium dataset, surpassing other models such as YOLOv5 and YOLOv8n, thereby confirming the broad applicability and generalization capabilities of our proposed improved model architecture. Additionally, evaluations on the refined URPC2019 dataset demonstrated leading performance, achieving an mAP@0.5 of 88.1%, which further confirms its superior performance on this dataset. These results highlight the model’s extensive applicability and generalization across various underwater datasets and provide valuable insights and contributions to future research in underwater target detection.

The structure of this document is as follows. Section 2 provides a review of the literature relevant to this field. The YOLOv8-MU model proposed in this study, along with the experimental analysis, is detailed in Section 3 and Section 4, respectively. Finally, Section 5 summarizes the contributions of this paper and outlines areas for future research.

## 2. Related Work

### 2.1. Object Detection

Object detection technology is mainly divided into two types: one-stage and two-stage object detection. Two-stage object detection first generates candidate region boxes and then classifies and regresses these boxes to determine the location, size, and category of the target. Common two-stage object detection algorithms include the R-CNN family, such as R-CNN [34] and Faster R-CNN [35]. Current research is focused on improving the models in the R-CNN family to make them more efficient and accurate. For example, Zeng et al. [36] proposed an underwater object detection algorithm based on Faster R-CNN and adversarial networks, enhancing the robustness and rapid detection capability of the detector. Song et al. [37] proposed an underwater object detection method based on an enhanced R-CNN detection framework to address challenges such as uneven illumination, low contrast, occlusion, and the camouflage of aquatic organisms in underwater environments. Hsia et al. [38] combined Mask R-CNN, data augmentation (DA), and discrete wavelet transform (DWT) to propose an intelligent retail product detection algorithm, improving the detection of overlooked objects.

One-stage object detection directly processes the entire image and simultaneously predicts the location, size, and category of the target through regression methods to improve the detection efficiency. Common one-stage object detection algorithms include the YOLO family, SSD, and RetinaNet. For example, the YOLO series of algorithms achieve rapid detection by dividing the image into grids and predicting the bounding boxes and classification confidence for each grid. The YOLO series has undergone multiple iterations and improvements. YOLOv1 [39] addressed the shortcomings of two-stage detection networks. YOLOv2 [40] added batch normalization layers after each convolutional layer and eliminated the use of dropout. YOLOv3 [41] introduced the residual module Darknet-53 and the feature pyramid network (FPN), resulting in significant improvements. The backbone network of YOLOv4 [42] is based on CSPDarknet53, using cross-stage partial connections (CSPs) to facilitate the information flow between different layers. YOLOv5 [43] introduced multi-scale prediction, automated hyperparameter optimization, and a more efficient model structure, leading to improvements in both speed and accuracy. YOLOv6 [44], YOLOv7 [45], and YOLOv8 [46] added many technologies on the basis of previous versions. There are also many improvements to the YOLO series to achieve more efficient detection performance. For example, Li et al. [47] proposed an improved YOLOv8 algorithm that integrates innovative modules from the real-time detection transformer (RT-DETR) to address the occlusion problem in underwater fish target detection. The algorithm, trained on an occlusion dataset using an exclusion loss function specifically designed for occlusion scenarios, significantly improves the detection accuracy. Additionally, SSD [48] uses a pyramid structure to classify and regress locations on multiple feature maps, making it more suitable for handling objects of different sizes. RetinaNet [49] introduces focal loss and a feature pyramid network to address the disparity between foreground and background classes, achieving higher accuracy.

In summary, two-stage object detection performs better in terms of accuracy but is slower in speed, whereas one-stage object detection has an advantage in speed but may lack accuracy. In practical applications, the choice between these methods depends on the specific requirements regarding the detection speed and accuracy.

### 2.2. Transformer

In the field of natural language processing (NLP), the Transformer model has become a mainstream technology that is widely recognized for its capabilities in understanding and generating text. Over time, researchers have begun to explore the application of Transformer architectures in the field of computer vision (CV), aiming to enhance the efficiency and accuracy of image-related tasks. In early attempts, Transformers were employed as enhanced decoders to optimize the model performance. For instance, Yang et al. [50] developed the TransPose model, which directly processed features extracted by convolutional neural networks (CNNs), to model the global relationships in images and effectively capture the dependencies between key points. On the other hand, Mao et al. [51] designed the Poseur method, utilizing lightweight Transformer decoders to achieve higher detection accuracy and computational efficiency.

Furthermore, Transformers have also been successfully applied to a broader range of image processing tasks. For example, the Vision Transformer (ViT) is a groundbreaking example that directly applies Transformer architectures to tasks such as image classification. Xu et al. [52] demonstrated the transferability of knowledge between different models and the flexibility of models through the ViTPose project. Recent research advances indicate that combining attention mechanisms from Transformers with object detection networks can lead to significant performance improvements. For instance, Wang et al. [53] integrated the SimAM attention module into the YOLO-BS network to improve the accuracy in detecting large coal blocks, helping to reduce congestion in underground conveyor systems. Similarly, BoTNet [54] introduced the BoT module with a self-attention mechanism, which optimizes and accelerates the training process of small networks by simulating the behavior of large networks, thereby effectively extracting and integrating features at different scales.

Based on these advanced observations and innovations, this study aimed to integrate attention mechanisms and Transformer modules into the YOLOv8 network architecture to further enhance the model’s performance in various object detection tasks. This introduction aimed to leverage the powerful global information modeling capabilities of Transformers to improve the efficiency and accuracy of image recognition and processing tasks.

### 2.3. SPP

In the research of machine vision and object recognition, the Spatial Pyramid Pooling (SPP) module and its improved versions, such as Spatial Pyramid Pooling Fast (SPPF), Simplified SPPF (SimSPPF), Atrous Spatial Pyramid Pooling (ASPP), Spatial Pyramid Pooling, Cross-Stage Partial Channel (SPPCSPC), and SPPFCSPC, have been widely utilized to improve the accuracy of object detection. These modules effectively address the problems caused by differences in input image sizes, avoiding image distortion. The initial concept of the SPP module was proposed by He et al. [55], aiming to overcome the challenge of inconsistent sizes. Subsequently, to further improve the processing speed, the SPPF [43] and SimSPPF [44] modules were developed successively. Additionally, Chen et al. introduced the ASPP module [56] in the DeepLabv2 semantic segmentation model, which enhances the recognition capability for multi-scale objects by capturing information at different scales through parallel dilated convolutions. The SPPCSPC module [45] achieves a performance improvement by optimizing the parameters and reducing the computational complexity, without expanding the receptive field.

In recent years, attention mechanisms have been introduced into object detection networks to enhance the models’ ability to detect small objects in complex scenes. For example, Wu et al. [57] proposed an effective multi-scale attention (EMA) mechanism based on multi-scale feature fusion, which automatically adjusts the weight distribution in the feature maps to focus more on key areas of the image. This is particularly effective in accurately identifying small objects in complex environments. Given this, this study aimed to integrate these improved SPP modules and attention mechanisms into the YOLOv8 network architecture, aiming to further optimize the performance of the network in various object detection tasks.

### 2.4. IoU Loss

In the research field of object detection, localization, and tracking, the precise regression of bounding boxes is crucial. In recent years, localization loss functions, represented by the intersection over union (IoU) loss [58] and its derivative versions [59,60,61,62], have played a central role in improving the accuracy of bounding box regression. These types of loss functions optimize the model by evaluating the overlap between the predicted bounding boxes and actual bounding boxes, effectively mitigating the impact of variations in the aspect ratio on the detection performance. However, the IoU loss has certain limitations. For instance, when the predicted box and the ground truth box do not overlap, the IoU value remains zero, failing to reflect the actual distance between them. Additionally, in cases where the IoU is the same, it cannot distinguish between positional differences.

To address these challenges, several studies have proposed various improvements to the IoU loss, including the Generalized IoU (GIoU), Distance IoU (DIoU), CIoU, Efficient IoU (EIoU), and Wise IoU (WIoU). GIoU loss overcomes the issue of traditional IoU calculation resulting in zero by introducing the concept of the minimum enclosing rectangle, although it may lead to smaller gradients and slower convergence in some scenarios [59]. DIoU loss enhances the model’s sensitivity to the position by considering the distance between the center points of the predicted and ground truth boxes, but it does not involve shape matching [60]. CIoU loss builds upon this by incorporating the difference in aspect ratio, although it may cause training instability in certain circumstances despite improving the shape-matching accuracy. EIoU loss balances the relationship between simple and hard samples by introducing separate consistency and focal losses, thereby enhancing the stability and efficiency of the model [61]. WIoU loss further enhances the model’s performance and robustness through a dynamic non-monotonic static focus mechanism (FM) [62].

In general, these variants of the IoU loss effectively improve the accuracy of bounding box regression and the robustness of the models by introducing mechanisms in loss calculation that consider the distance between the predicted and ground truth boxes, differences in the position center points, the consistency of the aspect ratios, and the handling of samples with varying difficulty levels. In practice, selecting the appropriate variant of the loss function tailored to specific object detection tasks is a key strategy in optimizing the detection performance.

## 3. Methodology

**Figure 1:**
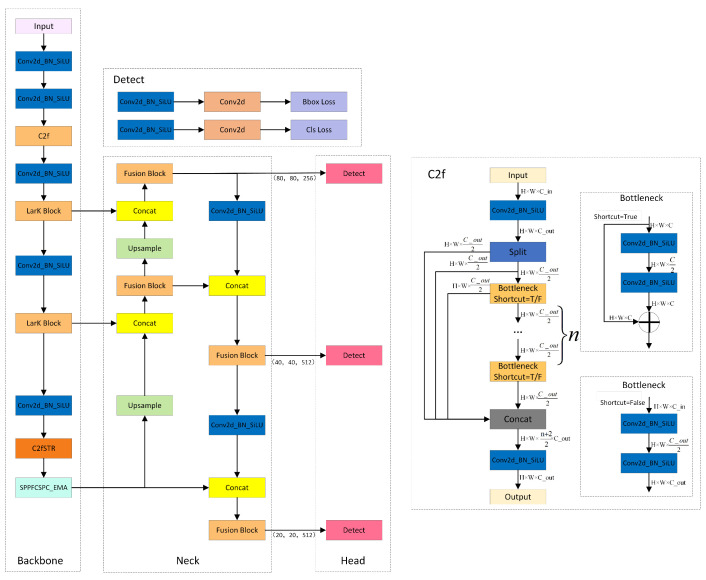
The structure of YOLOv8-MU. It consists of the backbone, neck, and head, including detailed structures of C2f and Detect [46].

While the YOLOv8 model has achieved significant progress in the field of object detection, it still exhibits certain limitations. Firstly, it adopts a larger network architecture, resulting in slower processing speeds compared to other models within the YOLO family. Secondly, for objects with limited feature information, the localization accuracy may not be sufficiently high. Furthermore, the absence of the consideration of inter-object relationships during the prediction process may lead to issues such as overlapping bounding boxes. Additionally, the utilization of fixed-scale anchor boxes may struggle to accommodate objects with varying aspect ratios, potentially resulting in object deformation. To address these issues, we designed YOLOv8-MU, as shown in Figure 1.

### 3.1. LarK Block

**Figure 2:**
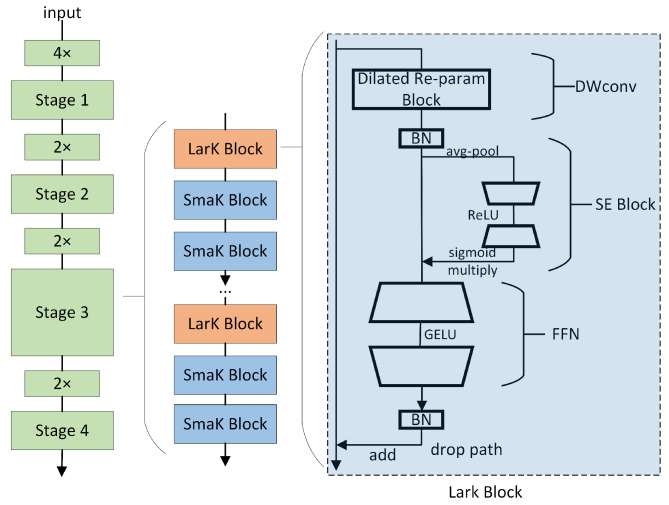
The structural design of UniRepLKNet. The LarK block consists of a dilated reparam block, SE block [63], feed-forward network (FFN), and batch normalization (BN) [64] layers. The only difference between the SmaK block and the LarK block is that the former uses a depth-wise 3 × 3 convolutional layer to replace the dilated reparam layer of the latter. Stages are connected by downsampling blocks, which are implemented by stride-2 dense 3 × 3 convolutional layers [30].

The convolutional neural network (ConvNet) with large kernels has shown remarkable performance in capturing sparse patterns and generating high-quality features, but there is still considerable room for exploration in its architectural design. While the Transformer model has demonstrated powerful versatility across multiple domains, it still faces some challenges and limitations in terms of computational efficiency, memory requirements, interpretability, and optimization. To address these limitations, we introduce the LarK block from UniRepLKNet into our model [30], as depicted in Figure 2. This block leverages the advantages of large kernel convolution to achieve a wider receptive field. By employing larger convolutional kernels, the LarK block can capture more contextual information without necessitating additional network layers. This represents a key advantage of large kernel convolution, enabling the network to capture richer features.

As illustrated in Figure 2, the block utilizing the dilated reparam block is referred to as the large kernel block (LarK block), while the block employing DWconv 3 × 3 is termed the small kernel block (SmaK block). The dilated reparam block is proposed based on equivalent transformations, with its core idea being the utilization of a non-sparse large kernel block (kernel size K = 9), combined with multiple sparse small kernel blocks (kernel sizes k are 5, 3, 3, 3), to enhance the feature extraction effectiveness. The sparsity rate r determines the distribution of non-zero elements within the convolution kernel, where a higher sparsity rate implies more zero elements within the kernel, aiding in reducing the computational complexity while maintaining the performance. For instance, to accommodate larger input sizes, when the large kernel K is increased to 13, the corresponding adjustment of the small kernel sizes and sparsity rates is made to be k = (5, 7, 3, 3, 3) and r = (1, 2, 3, 4, 5). This adjustment allows us to simulate an equivalent large convolutional layer with a kernel size of (5, 13, 7, 9, 11), effectively enhancing the feature extraction by integrating large kernel layers in this manner. We observe that, apart from capturing small-scale patterns, the ability to enhance a large kernel capturing sparse patterns may yield higher-quality features, aligning perfectly with the mechanism of dilated convolution [30]. From the perspective of sliding windows, dilated convolution layers with a dilation rate of d scan the input channels to capture spatial patterns, where the distance between each pixel of interest and its neighboring pixels is d − 1. Therefore, we adopt dilated convolution layers parallel to the large kernels and sum their outputs.

The large kernel block is primarily integrated into the middle and upper layers of the model to enhance the depth and expressive capability of the model when using large kernel convolutional layers. This enhancement is achieved by stacking multiple SE blocks to deepen the model. The squeeze-and-excitation (SE) block compresses all channels of the feature map into a single vector through a global compression operation, which contains global contextual information about the features. Then, this vector is activated through a fully connected layer and a sigmoid activation function to restore the number of channels to match the input features. This activation vector is multiplied element-wise with the original feature map, thereby enhancing or suppressing certain channels in the feature map. The SE block can enhance the model’s feature expression capability, especially in the early stages, particularly when there is a lack of sufficient contextual information.

### 3.2. C2fSTR

**Figure 3:**
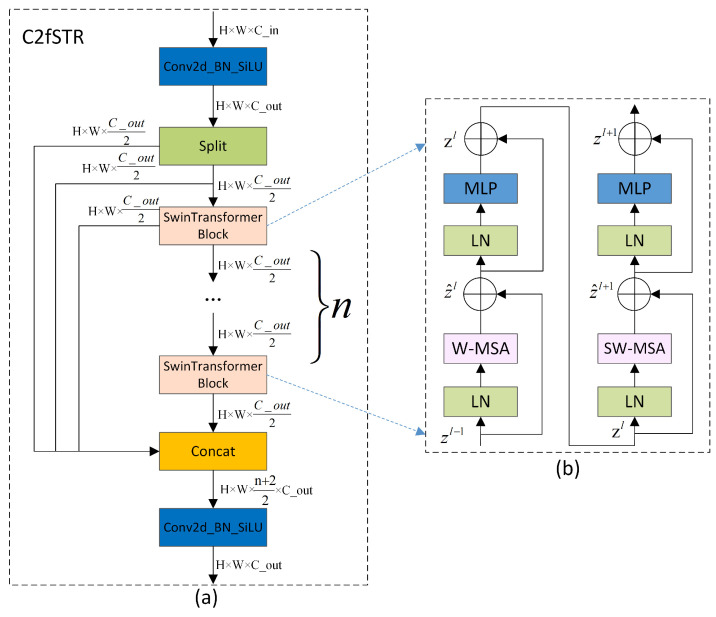
(**a**) The structure of the C2fSTR. (**b**) Two consecutive Swin Transformer blocks (represented by Equation (1)). W-MSA and SW-MSA are multi-head self-attention modules, employing regular and shifted window configurations, respectively [28].

The proposed C2fSTR in this paper modifies the original YOLOv8 architecture’s C2f module using the Swin Transformer block [28]. Compared to the original C2f module, the modified C2fSTR module facilitates better interactions between strong feature maps and fully utilizes the target background information, thereby enhancing the accuracy and robustness of object detection under complex background conditions. Figure 3a illustrates the structure of the C2fSTR.

The C2fSTR consists of two modules. One is the Conv module, which consists of a Conv2d with a kernel size of 1 × 1 and a stride of 1, followed by batch normalization and the Silu activation function. The role of the convolution module is to reduce the length and width of the feature map while expanding the dimensionality. The other module is the Swin Transformer block, which comprises a linear layer (LN), a shifted window multi-head self-attention (SW-MSA), and a feed-forward MLP (MLP). The structure includes some Swin Transformer modules. The function of the Swin Transformer block is to expand the scope of the information interaction without increasing the number of parameters by restricting the attention computations to be within each window. Its structure is illustrated in Figure 3b.

Traditional Transformers typically compute the attention globally, leading to high computational complexity. The computational complexity of the multi-head attention mechanism is proportional to the square of the size of the feature map. To reduce the computational complexity of the multi-head attention mechanism and expand the range of the information interaction, in the Swin Transformer, the feature map is divided into windows. Each window undergoes window-based multi-head self-attention computation followed by shifted window-based multi-head self-attention computation, enabling mutual communication between windows [65]. The computation of consecutive Swin Transformer blocks is shown in Equation (1):

(1) $$\begin{array}{ccc} {\hat{z}}^{l} & = & \mathrm{W-MSA}\left(\mathrm{LN}\left(z^{l-1}\right)\right)+z^{l-1},\\ z^{l} & = & \mathrm{MLP}\left(\mathrm{LN}\left({\hat{z}}^{l}\right)\right)+{\hat{z}}^{l},\\ {\hat{z}}^{l+1} & = & \mathrm{SW-MSA}\left(\mathrm{LN}\left(z^{l}\right)\right)+z^{l},\\ z^{l+1} & = & \mathrm{MLP}\left(\mathrm{LN}\left({\hat{z}}^{l+1}\right)\right)+{\hat{z}}^{l+1}. \end{array}$$

where ${\hat{z}}^{l}$ and $z^{l}$ represent the output features of the (S)W-MSA and MLP modules of block *l*, respectively, and W-MSA and SW-MSA represent window-based multi-head self-attention using regular and shifted window partitioning configurations, respectively.

When employing the window-based multi-head self-attention (W-MSA) module, self-attention calculations are conducted solely within individual windows, thereby preventing information exchange between separate windows. To address this limitation, the model incorporates the shifted window multi-head self-attention (SW-MSA) module, which is an offset adaptation of the W-MSA. However, the shifted window partitioning approach introduces another issue: it results in the proliferation of windows, and some of these windows are smaller than standard windows. For instance, a window comprising 2 × 2 patches may expand to encompass 3 × 3 patches, more than doubling the number of windows, which may consequently lead to an increase in parameters. To resolve this issue, a cyclic shift along the top-left direction is proposed. This method involves cyclically shifting the input features, enabling the windows within a batch to consist of discontinuous sub-windows, thereby maintaining a constant number of windows. Thus, although the shifted window strategy intrinsically increases the number of windows, the cyclic shift approach effectively mitigates this issue by ensuring the stability of the window count.

In this way, by confining the attention computations to each window, the Swin Transformer enhances the model’s focus on local features, thereby augmenting its ability to model local details. However, object recognition and localization in images depend on the feature information of the global background. The information interaction in the Swin Transformer is limited to individual windows and shifted windows, capturing only local details of the target, while global background information is difficult to obtain [66]. To achieve a more extensive information interaction and simultaneously obtain both global background and local detail information, we apply the Swin Transformer block to C2f, replacing the Darknet bottleneck and forming the C2fSTR feature backbone system. This combined strategy enables a comprehensive information interaction, effectively capturing rich spatial details and significantly improving the model’s accuracy in object detection in complex backgrounds.

### 3.3. SPPFCSPC_EMA

**Figure 4:**
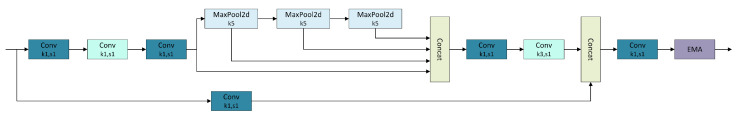
The structure of SPPFCSPC_EMA. SPPFCSPC performs a series of convolutions on the feature map, followed by max-pooling and fusion over four receptive fields (one 3 × 3 and three 7 × 7). After further convolution, it is fused with the original feature map and finally combined with EMA to form the SPPFCSPC_EMA module (Conv: convolution; MaxPool2d: max pooling) [68].

As shown in Figure 4, YOLOv8-MU replaces the SPPF module in YOLOv8 with the SPPFCSPC module and introduces multiple convolutions and concatenation techniques to extract and fuse features at different scales, expanding the receptive field of the model and thereby improving the model’s accuracy. Additionally, we have introduced the EMA module, whose parallel processing and self-attention strategy significantly improve the model’s performance and optimize the feature representation [67]. By combining the SPPFCSPC and EMA modules to form the SPPFCSPC_EMA module, not only are the model’s accuracy, efficiency, and robustness enhanced, but the model’s performance is further improved while maintaining its efficiency.

The SPPFCSPC module integrates two submodules: SPP and fully connected spatial pyramid convolution (FCSPC) [69]. SPP, as a pooling layer, can handle input feature maps of different scales, effectively detecting both small and large targets. FCSPC is an improved convolutional layer aimed at optimizing the representation of the feature maps to enhance the detection performance. By performing multi-scale spatial pyramid pooling on the input feature map, the SPP module captures information about targets and scenes at different scales [55]. Subsequently, the FCSPC module convolves the feature maps of different scales output by the SPP module and divides the input feature map into blocks. These blocks are pooled and concatenated, followed by convolution operations, to enhance the model’s receptive field and retain key feature information, thereby improving the model’s accuracy [69]. The SPPFCSPC module is an optimization of SPPCSPC based on the SPPF concept, reducing the computational requirements for the pooling layer’s output by connecting three independent pooling operations and improving the speed and detection accuracy of dense targets without changing the receptive field [68]. The results produced using this pooling method are comparable to those obtained using larger pooling kernels, thus optimizing the training and inference speeds of the model. The calculation formula for the pooling part is shown in Equation (2):

(2) $$\begin{array}{ccc} S_{1}\left(R\right) & = & {\mathrm{MaxPool}}_{k=5}^{p=2}\left(R\right)\\ S_{2}\left(S_{1}\right) & = & {\mathrm{MaxPool}}_{k=5}^{p=2}\left(S_{1}\right)\\ S_{3}\left(S_{2}\right) & = & {\mathrm{MaxPool}}_{k=5}^{p=2}\left(S_{2}\right)\\ S_{4} & = & S_{1}⊛S_{2}⊛S_{3} \end{array}$$

where *R* represents the input feature layer, $S_{1}$ represents the pooling layer result of the smallest pooling kernel, $S_{2}$ represents the pooling layer result of the medium-sized pooling kernel, $S_{3}$ represents the pooling layer result of the largest pooling kernel, $S_{4}$ represents the final output result, and ⊛ represents tensor concatenation.

**Figure 5:**
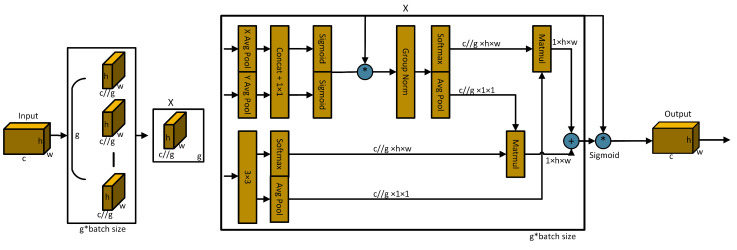
Schematic diagram of EMA. Here, ‘g’ denotes grouping, ‘X Avg Pool’ represents 1D horizontal global pooling, ‘Y Avg Pool’ represents 1D vertical global pooling, and ‘*’ indicates reparameterization [67].

The EMA [67] mechanism employs three parallel pathways, including two 1 × 1 branches and one 3 × 3 branch, to enhance the processing capability for spatial information. In the 1 × 1 branches, global spatial information is extracted through two-dimensional global average pooling, and the softmax function is utilized to ensure computational efficiency. The output of the 3 × 3 branch is directly adjusted to align with the corresponding dimensional structure before the joint activation mechanism, which combines channel features, as shown in Equation (3). An initial spatial attention map is generated through matrix dot product operations, integrating spatial information of different scales within the same processing stage. Furthermore, the 2D global average pooling embeds global spatial information into the 3 × 3 branch, producing a second spatial attention map that preserves precise information on the spatial location. Finally, the output feature maps within each group are further processed through the sigmoid function [70]. As illustrated in Figure 5, the design of EMA aims to assist the model in capturing the interactions between features at different scales, thereby enhancing the performance of the model.

(3) $$\begin{array}{ccc} z_{c} & = & \frac{1}{H\times W}\sum_{j}\sum_{i}x_{c}\left(i,j\right) \end{array}$$

Here, $z_{c}$ represents the output related to the *c*-th channel. The primary purpose of this output is to encode global information, thereby capturing and modeling long-range dependencies.

Therefore, the overall formula for the SPPFCSPC_EMA module is as shown in Equation (4):

(4) $$\begin{array}{ccc} z_{c} & = & \frac{1}{H\times W}\sum_{j}\sum_{i}S_{4}\left(i,j\right) \end{array}$$

### 3.4. Fusion Block

**Figure 6:**
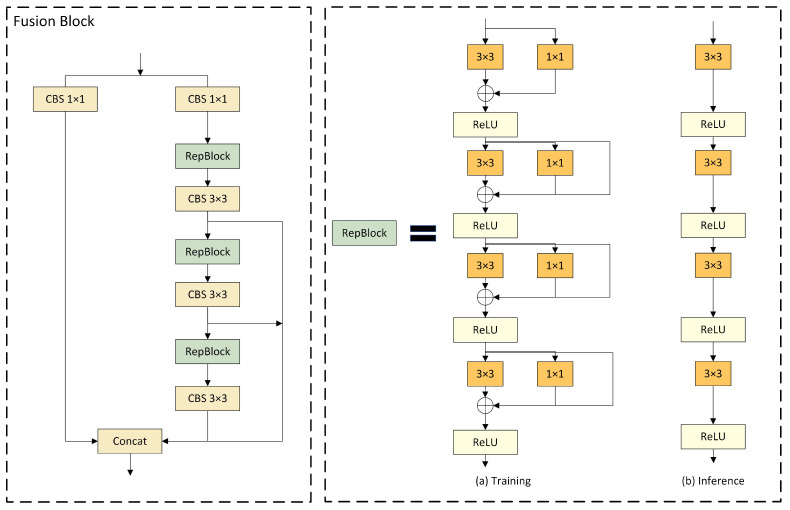
Structural diagram of the fusion block, which includes a schematic diagram of the RepBlock. (**a**) represents the model structure used during training, and (**b**) represents the model structure used during inference [73].

DAMO-YOLO has improved the efficiency of node stacking operations and optimized feature fusion by introducing a specially designed fusion block. Inspired by this, we replaced the C2f module in the neck network with the fusion block to enhance the fusion capability for multi-scale features. As illustrated in Figure 6, the architecture of the fusion block commences with channel number adjustment on two parallel branches through 1 × 1 CBS, followed by the incorporation of the concept of feature aggregation from the efficient layer aggregation network (ELAN) [71] into the subsequent branch, composed of multiple RepBlocks and 3 × 3 CBS. This design leverages strategies such as CSPNet [72], the reparameterization mechanism, and multi-layer aggregation to effectively promote rich gradient flow information at various levels. Furthermore, the introduction of the reparameterized convolutional module significantly enhances the performance.

Four gradient-path fusion blocks are utilized in the model, each splitting the input feature map into two streams. One stream is directly connected to the output, while the other undergoes channel reduction, cross-level edge processing, and convolutional reparameterization before further dividing into three gradient paths from this stream. Ultimately, all paths are merged into the output feature map. This design segments the gradient flow paths, introducing variability in the gradient information as it moves through the network, effectively facilitating a richer flow of gradient information.

As for Figure 6, the RepBlock is designed to employ different network structures during the training and inference phases through the use of reparameterization techniques, thereby achieving efficient model training and rapid inference speeds [73]. Following the recommendations of RepVGG, we optimized the parameter structure, clearly segregating the multi-branch used during the training phase from the single branch used during the inference phase. During the training process, the RepBlock adopts a complex structure containing multiple parallel branches, which extract features through 3 × 3 convolutions, 1 × 1 convolutions, and batch normalization (BN). This design is intended to enhance the representational capacity of the model. During inference, these multi-branch structures are converted into a single, more streamlined 3 × 3 convolutional layer through structural reparameterization, eliminating the branch structure to increase the inference speed and reduce the memory consumption of the model.

The conversion from a multi-branch to a single-branch architecture is primarily motivated by three considerations. Firstly, from the perspective of speed, the models reparameterized for inference demonstrate a significant acceleration in inference speed. This not only expedites the model inference process but also enhances the practicality of model deployment. Secondly, regarding memory consumption, the multi-branch model necessitates the allocation of memory individually for each branch to store its computational results, leading to substantial memory usage. Adopting a single-path model significantly reduces the demand for memory. Lastly, in terms of model flexibility, the multi-branch model is constrained by the requirement that the input and output channels for each branch remain consistent, posing challenges to model modification and optimization. In contrast, the single-path model is not subject to such limitations, thereby increasing the flexibility of model adjustments.

### 3.5. MPDIOU

Although they consider multiple factors, existing boundary box regression loss functions, such as CIoU, may still exhibit inaccurate localization and blurred boundary issues when dealing with complex scenarios where the target boundary information is unclear, affecting the regression accuracy. Given the intricate underwater environment and limited lighting conditions, the boundary information of target objects is often inadequate, posing challenges that prevent traditional loss functions from adapting effectively. Inspired by the geometric properties of a horizontal rectangle, Ma et al. [33] designed a novel boundary box regression loss function based on the minimum point distance $L_{\mathrm{MPD}IoU}$. We incorporated this function, referred to as MPDIoU, into our model to evaluate the similarity between the predicted and ground truth boundary boxes. Compared to existing loss functions, MPDIoU not only better accommodates blurred boundary scenarios and enhances the object detection accuracy but also accelerates the model’s convergence and reduces the redundant computational overhead, thereby improving the localization and boundary precision for underwater organism detection.

The calculation process of MPDIoU is as follows. Assume that $\left(x_{1}^{\mathrm{gt}},y_{1}^{\mathrm{gt}}\right)$ and $\left(x_{2}^{\mathrm{gt}},y_{2}^{\mathrm{gt}}\right)$ represent the coordinates of the top-left and bottom-right points of the ground truth box, respectively, and $\left(x_{2}^{\mathrm{pd}},y_{2}^{\mathrm{pd}}\right)$ and $\left(x_{1}^{\mathrm{pd}},y_{1}^{\mathrm{pd}}\right)$ represent the coordinates of the top-left and bottom-right points of the predicted box, respectively. Parameters w and h represent the width and height of the input image, respectively. The formulas for the ground truth box and the predicted box are $d_{1}^{2}={\left(x_{1}^{pd}-x_{1}^{gt}\right)}^{2}+{\left(y_{1}^{pd}-y_{1}^{gt}\right)}^{2}$ and $d_{2}^{2}={\left(x_{2}^{pd}-x_{2}^{gt}\right)}^{2}+{\left(y_{2}^{pd}-y_{2}^{gt}\right)}^{2}$, respectively.

Subsequently, the final $L_{\mathrm{MPD}IoU}$ can be calculated using Equations (5) and (6) based on $d_{1}$ and $d_{2}$.

(5) $$\begin{array}{ccc} \mathrm{MPD}IoU & = & \frac{A\cap B}{A\cup B}-\frac{d_{1}^{2}}{w^{2}+h^{2}}-\frac{d_{2}^{2}}{w^{2}+h^{2}} \end{array}$$

(6) $$\begin{array}{ccc} L_{\mathrm{MPD}IoU} & = & 1-\mathrm{MPD}IoU \end{array}$$

The MPDIoU loss function optimizes the similarity measurement between two bounding boxes, enabling it to adapt to scenarios involving both overlapping and non-overlapping bounding box regression. Moreover, all components of the existing bounding box regression loss functions can be represented using four-point coordinates, as shown in Equations (7)–(9).

(7) $$\begin{array}{ccc} \left|C\right| & = & \left(\max\left(x_{2}^{gt},x_{2}^{pd}\right)-\min\left(x_{1}^{gt},x_{1}^{pd}\right)\right)\times \left(\max\left(y_{2}^{gt},y_{2}^{pd}\right)-\min\left(y_{1}^{gt},y_{1}^{pd}\right)\right) \end{array}$$

(8) $$\begin{array}{c} x_{c}^{gt}=\frac{x_{1}^{gt}+x_{2}^{gt}}{2},\;y_{c}^{gt}=\frac{y_{1}^{gt}+y_{2}^{gt}}{2},\;x_{c}^{pd}=\frac{x_{1}^{pd}+x_{2}^{pd}}{2},\;y_{c}^{pd}=\frac{y_{1}^{pd}+y_{2}^{pd}}{2} \end{array}$$

(9) $$\begin{array}{c} w_{gt}=\left|x_{2}^{gt}-x_{1}^{gt}\right|,\;h_{gt}=\left|y_{2}^{gt}-y_{1}^{gt}\right|,\;w_{pd}=\left|x_{2}^{pd}-x_{1}^{pd}\right|,\;h_{pd}=\left|y_{2}^{pd}-y_{1}^{pd}\right| \end{array}$$

where |C| represents the area of the smallest bounding rectangle encompassing both the ground truth and predicted boxes. The center coordinates of the ground truth and predicted boxes are denoted by $\left(x_{c}^{\mathrm{gt}},y_{c}^{\mathrm{gt}}\right)$ and $\left(x_{c}^{\mathrm{pd}},y_{c}^{\mathrm{pd}}\right)$, respectively, while their widths and heights are also represented. Through Equations (7)–(9), we can calculate the non-overlapping area, the distance between the center points, and the deviation in width and height. This method not only ensures comprehensiveness but also simplifies the computational process. Therefore, in the localization loss part of the YOLOv8-MU model, we choose to use the MPDIoU function to calculate the loss, to enhance the model’s localization accuracy and efficiency.

## 4. Experimental Details

### 4.1. Benchmark Testing and Implementation Details

#### 4.1.1. Dataset

**Figure 7:**
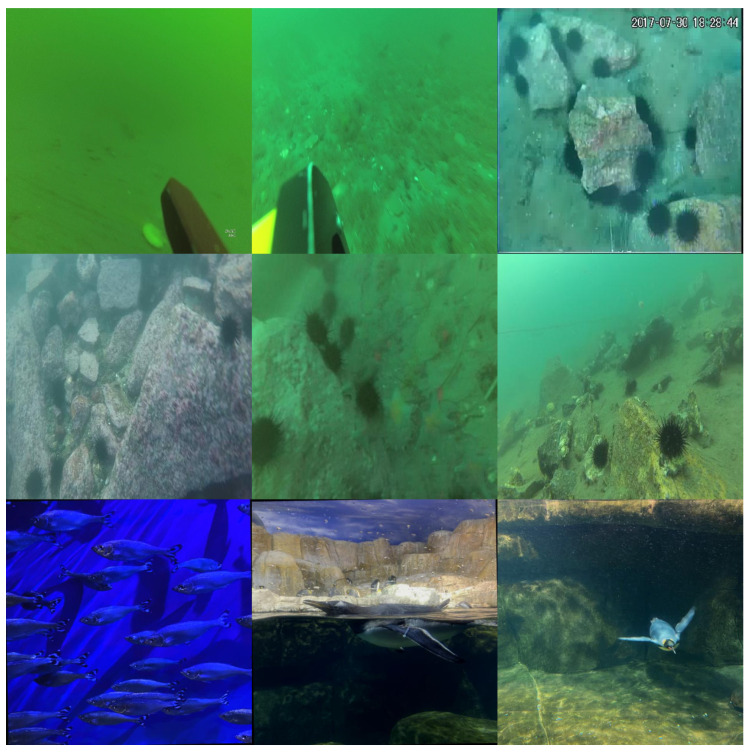
Example images from the URPC2019, URPC2020, and Aquarium datasets.

In this study, the dataset used to validate the effectiveness of our optimized model was URPC2019 (http://www.urpc.org.cn/index.html, accessed on 15 June 2023), a publicly available dataset for underwater object detection. It includes five different categories of aquatic life, Echinus, starfish, Holothurian, scallops, and waterweeds, with a total of 3765 training samples and 942 validation samples. Examples of the dataset’s images are shown in the first row of Figure 7. Simultaneously, we conducted experiments on the URPC2019 dataset (in the absence of waterweeds) and the refined URPC2019 dataset to further demonstrate the superior detection accuracy of our proposed improved model. Additionally, we performed detection experiments on the URPC2020 (http://www.urpc.org.cn/index.html, accessed on 15 June 2023) dataset. Similar to URPC2019, URPC2020 is an underwater dataset, but it differs in that it contains only four categories, Echinus, starfish, Holothurian, and scallops, with a total of 4200 training samples and 800 validation samples. The second row of Figure 7 displays examples of images from this dataset. Finally, we conducted experiments on the Aquarium dataset, which differs from the URPC series in terms of the types of seabed substrates. The Aquarium dataset, provided by Roboflow (https://universe.roboflow.com/brad-dwyer/aquarium-combined/3, accessed on 20 April 2024), encompasses various categories, such as fish, jellyfish, penguins, puffins, sharks, starfish, and minks. Additionally, the dataset includes augmented versions, incorporating rotations and flips, totaling 4670 images, comprising 4480 training images, 63 testing images, and 127 validation images. Examples of images from this dataset are illustrated in the third row of Figure 7. Through experiments conducted on these three datasets, we aimed to validate the feasibility and extensive applicability of our model.

#### 4.1.2. Environment Configuration and Parameter Settings

The experiments in this study were conducted on the Ubuntu 20.04, utilizing the PyTorch 1.11.0 deep learning framework. The experimental setup included the parallel computing platform and programming model developed by NVIDIA (Santa Clara, CA, USA), the Python 3.8 programming language, and server processors released by Intel. The performance of different GPUs and the size of the RAM significantly impact the experimental results. Therefore, we maintained a consistent experimental environment throughout our entire experimental process. The specific configuration is shown in Table 1.

To enhance the persuasiveness of the experiments, we conducted experiments based on the original YOLOv8 model, during which a series of parameter adjustments were made and multiple experimental tests were conducted. Ultimately, we determined that some of the main hyperparameters for all experiments would adopt the same settings as shown in Table 1. A larger batch size can speed up training, so we set it to 16. In terms of loss calculation, we continued YOLOv8’s approach of combining the classification loss, bounding box regression loss, and distribution focal loss, with the weights of the three losses being 7.5, 0.5, and 1.5, respectively, to optimize the model. In addition, momentum and weight decay were important hyperparameters for the optimization of the model, with the detailed settings available in Table 2.

#### 4.1.3. Evaluation Criteria

Evaluating the quality of YOLO models requires a comprehensive consideration of speed, accuracy, applicability, robustness, and cost, among other factors, with varying focus points in different use scenarios. For the URPC series datasets, this study primarily focuses on the accuracy of the improved YOLOv8 model. We assess the model’s accuracy by calculating and comparing the average precision (AP) for each class and the mean average precision (mAP). Additionally, we examine the impact of floating point operations (FLOPs) and the number of parameters (Para) on the model accuracy to verify the superiority of our improved YOLOv8 model.

The calculation of the AP value is related to the calculation and integration of the precision–recall curve. First, it is necessary to calculate the precision and recall values using Equations (10) and (11), where TP, FP, and FN represent true positive, false positive, and false negative. True positive is the number of positive samples predicted as positive by the model; false positive is the number of negative samples predicted as positive by the model; false negative is the number of positive samples predicted as negative by the model. Subsequently, the average precision for each category is calculated according to Equation (12). To reflect the performance of the model on the entire dataset, the mAP’s value is calculated according to Equation (13). In the calculation of the mAP, we take the value at an IoU of 0.5 and write it as mAP@0.5, which means that the detection is considered successful only when the intersection part of the true box and our predicted box is greater than 50%.

(10) $$\begin{array}{ccc} \mathrm{Precision} & = & \frac{TP}{\left(TP+FP\right)} \end{array}$$

(11) $$\begin{array}{ccc} \mathrm{Recall} & = & \frac{TP}{\left(TP+FN\right)} \end{array}$$

(12) $$\begin{array}{ccc} AP & = & \int_{0}^{1}P\left(R\right)\;dR \end{array}$$

(13) $$mAP=\frac{1}{N}\sum_{i=1}^{n}AP_{i}$$

### 4.2. Comparative Experiments

#### 4.2.1. Experiments on URPC2019

**Figure 8:**
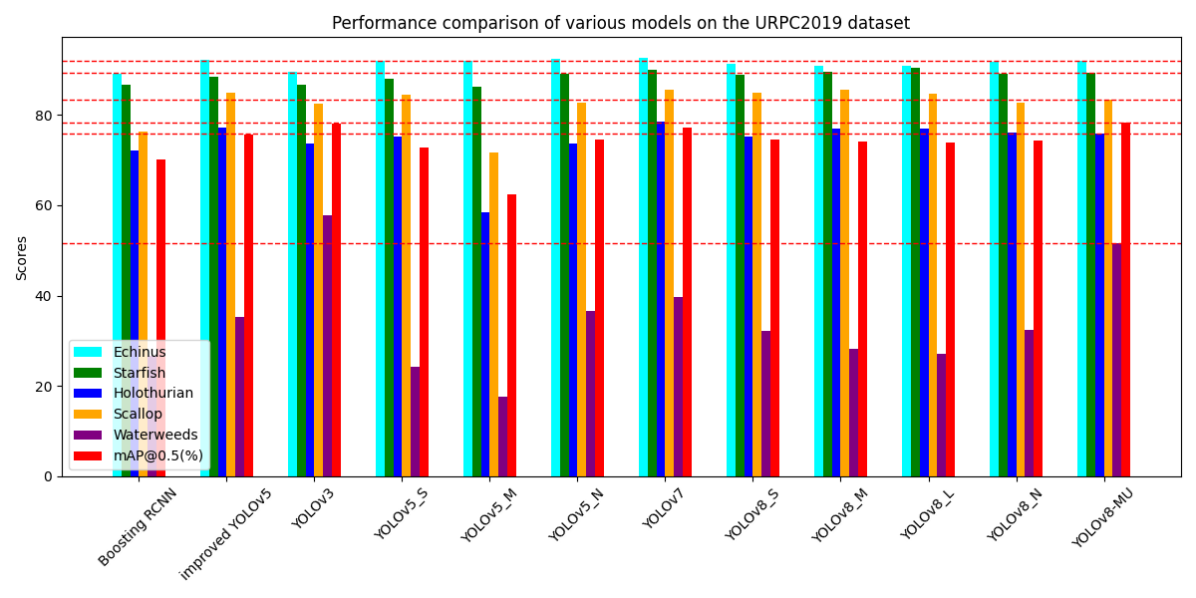
Performance comparison of various models on the URPC2019 dataset.The red line represents Scores of YOLOv8-MU.

We first conducted a literature search or experiments on the performance of various models on the URPC2019 dataset, including the Boosting R-CNN [37] model, which introduces the idea of reinforcement learning to improve Faster R-CNN [35], the YOLOv3 model, the YOLOv5 series models, the improved YOLOv5 [73], the YOLOv7 model, the YOLOv8 series models, and our optimized YOLOv8 model. The experimental data are shown in Table 3. We also plotted a bar graph, as shown in Figure 8, to provide a more intuitive comparison of the performance of each model.

After our observation and analysis, we find that the optimized model performs better than the other models, especially since the optimization of the AP values of each category is more obvious. Particularly in the detection of the waterweeds category, the data performance is quite good, with an AP value increase of 25.2% compared to the traditional Boosting R-CNN model. The AP value is also only slightly lower than that of the YOLOv3 model compared to the YOLO series models, and there is an increase of nearly 20% compared to the baseline model, YOLOv8n. This indicates that the improved YOLOv8 model has overcome the difficulties faced by other models in detecting the waterweeds category, demonstrating a unique advantage in enhancing the AP value for the individual category of waterweeds.

Furthermore, upon analyzing the mAP@0.5 values, we find that the YOLOv8-MU model also demonstrates superior performance in terms of the overall dataset detection accuracy. The mAP@0.5 of YOLOv8-MU is the highest in Table 3, namely 78.4%, which is 8.2% higher than that of the traditional Boosting R-CNN model, 2.7% higher than that of the improved YOLOv5 [73], and 4% higher than that of the baseline model, YOLOv8n. It is closest to the YOLOv3 model but shows an improvement. The main reason is that although the AP value of YOLOv8-MU in the waterweeds category is lower than that of YOLOv3, YOLOv8-MU has higher detection accuracy in the remaining four categories compared to YOLOv3. This also verifies the effectiveness of YOLOv8-MU in improving the overall detection accuracy on the URPC2019 dataset.

**Figure 9:**
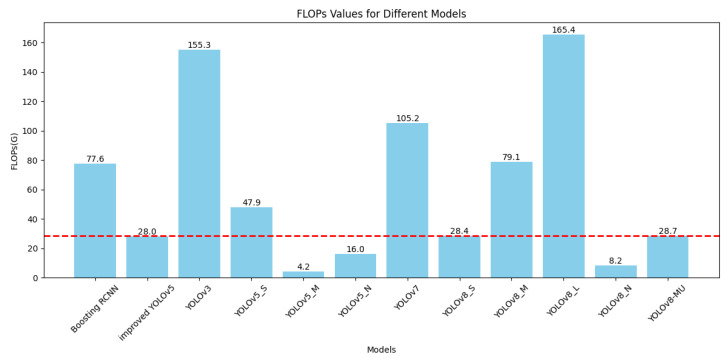
Bar graph comparison of FLOPs for various models on the URPC2019 dataset. The red line represents FLOPs of YOLOv8-MU.

**Figure 10:**
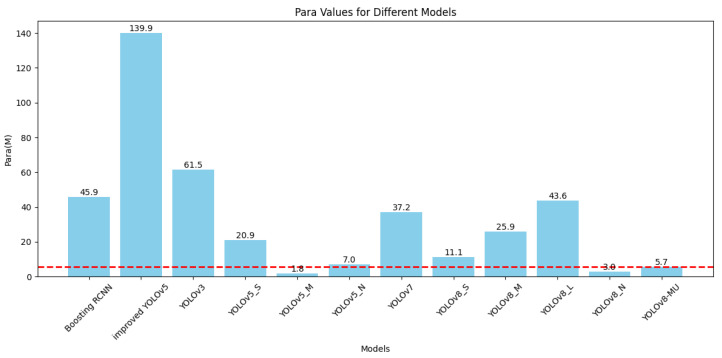
Bar graph comparison of the number of parameters for various models on the URPC2019 dataset.

In deep learning models, a relatively low number of parameters and FLOPs can reduce the model’s computational complexity and size, enhancing its performance and applicability in practical applications. For this reason, we specifically plotted the bar graphs shown in Figure 9 and Figure 10 based on Table 3 to compare the number of parameters and FLOPs among various models. It can be seen that although the number of parameters and FLOPs in our optimized model, YOLOv8-MU, has increased compared to the baseline model, YOLOv8n, they are still reduced compared to other models. This proves that our model achieves the effect of being lightweight.

**Figure 11:**
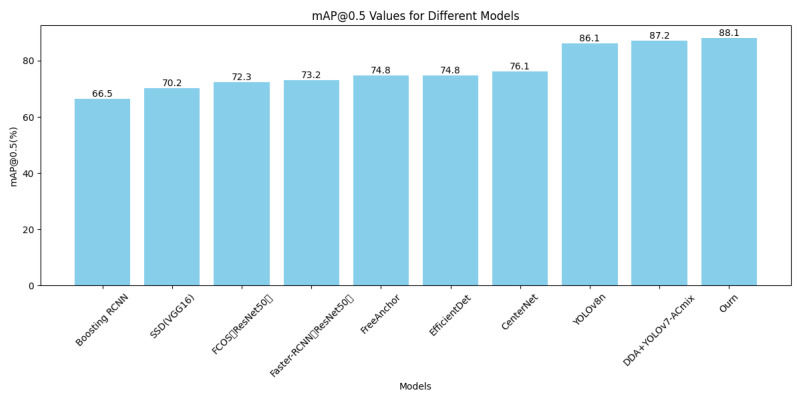
Map values of different models on the URPC2019 dataset with the waterweed category removed.

Additionally, experiments were conducted on both the URPC2019 dataset (without waterweeds) and the refined URPC2019 dataset, to compare our proposed YOLOv8-MU model with several recent underwater object detection methods, as shown in Table 4. We also drew bar charts based on maps of different models, as shown in Figure 11, to provide a more intuitive comparison of our model with other models. The URPC2019 refined dataset comprises a total of 4757 images, while the URPC2019 dataset (without waterweeds) contains 4707 images. Although the total number of images differs, our model demonstrates superior detection accuracy even with fewer photos, further highlighting the superiority of our model in terms of detection precision.

**Figure 12:**
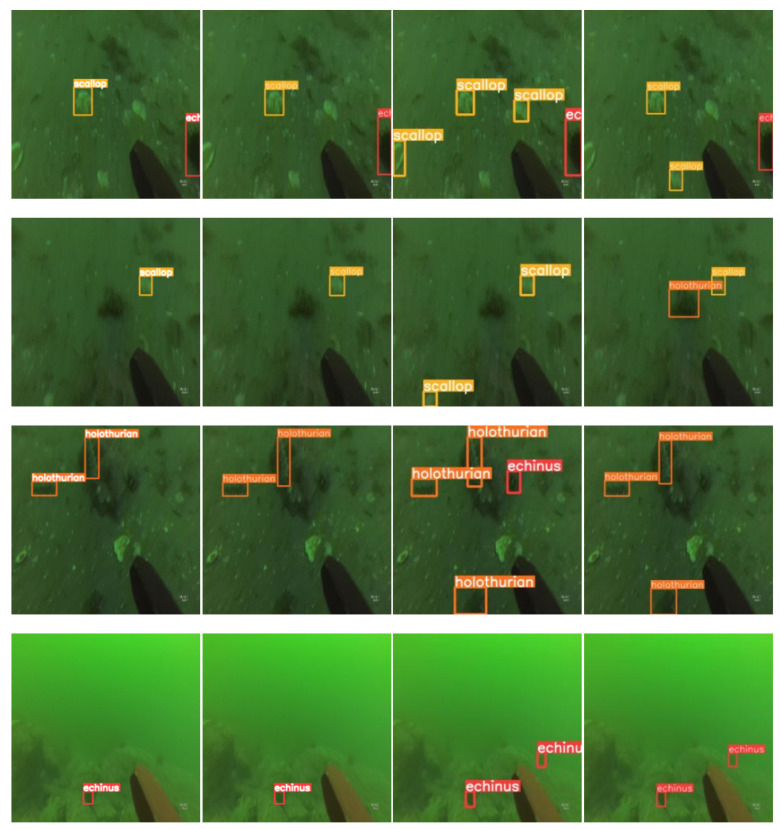

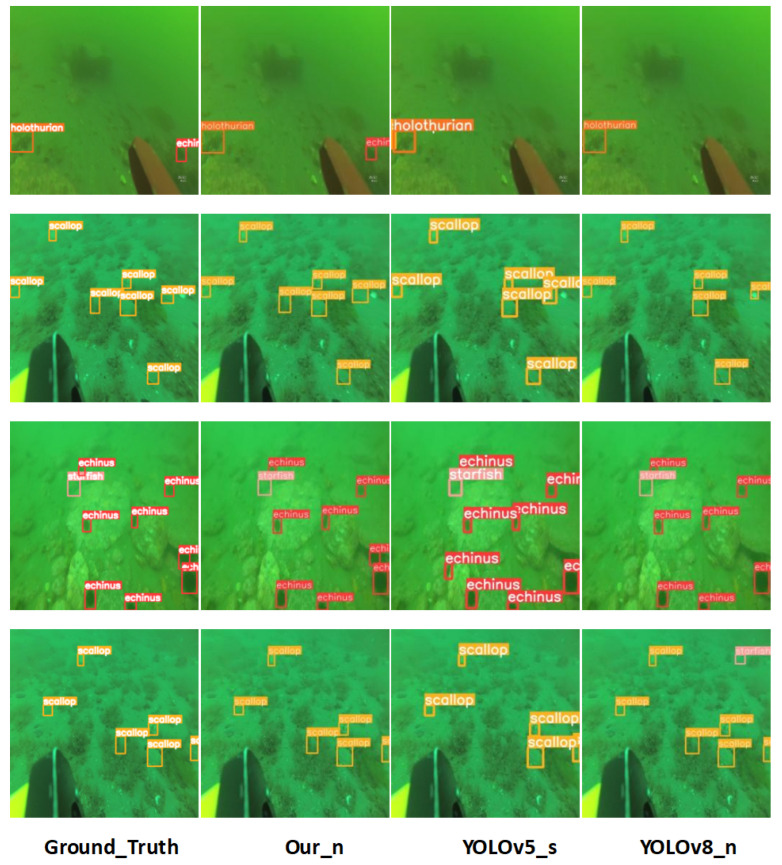
Comparison of target detection results between different models.

To more intuitively demonstrate the superiority of our optimized YOLOv8 model’s detection performance, we extracted and compared the detection results of different models on the URPC2019 dataset, as shown in Figure 12. Our model outperformed other models in both precision and recall. As can be seen clearly in rows 1 to 4, our optimized model did not detect any targets beyond the ground truth, indicating that our model has high precision. In the results for the images in rows 5 to 8, both YOLOv5s and YOLOv8n exhibit the same issue, failing to detect all targets in the ground truth and missing some targets, while our model exhibits high recall. This sufficiently demonstrates the effectiveness of our optimized YOLOv8 model in detection on the URPC2019 dataset.

#### 4.2.2. Experiments on URPC2020

**Figure 13:**
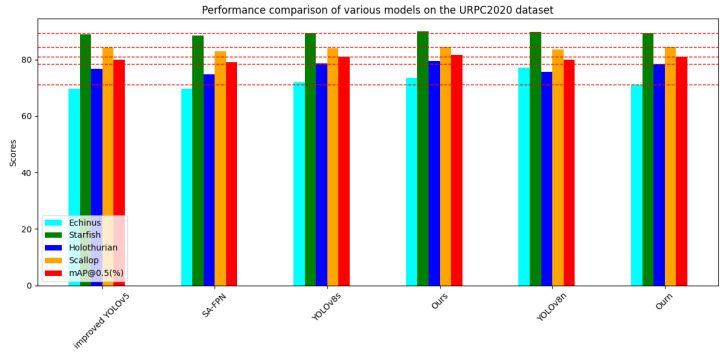
Performance comparison of various models on the URPC2020 dataset.The red line represents Scores of Ourn.

**Figure 14:**
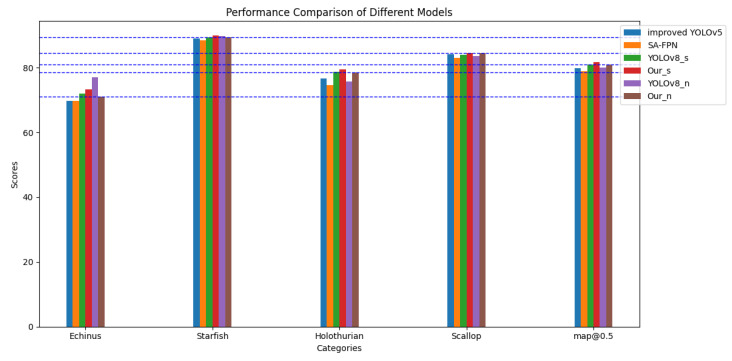
Performance comparison of various models on the URPC2020 dataset.

On the URPC2020 dataset, which is part of the same series as URPC2019, we also conducted a series of experiments. The results are presented in Table 5; based on these results, we plotted bar graphs with different horizontal axes, as shown in Figure 13 and Figure 14. We observed that the URPC2020 dataset, unlike URPC2019, has only four biological categories and is missing the Waterweeds category, which leads to high AP values for a single category, resulting in a small improvement in the detection performance relative to other models but an improvement that is sufficient to reflect the advantages of our model. We compared the experimental results of the improved YOLOv5 [73], SA-SPPN [79], and YOLOv8n with our model, Our_n, and found that the mAP@0.5 score of our improved model was higher than those of the other models. Additionally, we compared YOLOv8s with ours to demonstrate the high efficiency of our improved model in terms of detection accuracy.

#### 4.2.3. Experiments on Aquarium

**Figure 15:**
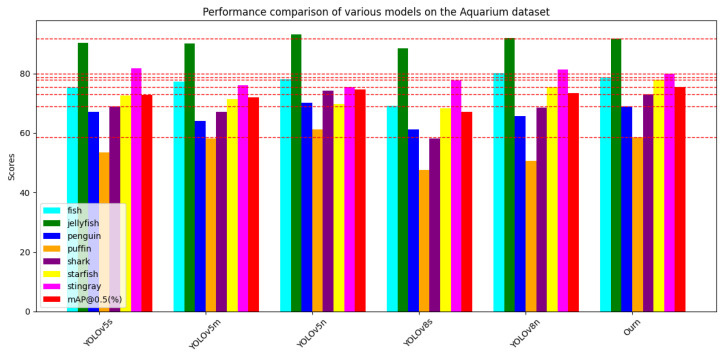
Performance comparison of various models on the Aquarium dataset.

To validate the extensive applicability of our enhanced model across various seabed substrates and diverse datasets, we conducted supplementary experiments on the Aquarium dataset. The results are detailed in Table 6. Additionally, we visualized the performance metrics using bar charts with distinct horizontal axes, as depicted in Figure 15. Notably, on the Aquarium dataset, we observed a deficiency in robust performance regarding the AP values for the puffin category, resulting in a relatively minor enhancement in the detection performance for this specific category. Nonetheless, in comparison to the alternative models, our proposed YOLOv8-MU not only achieves higher AP values in detecting the puffin category but also outperforms other models in the overall mAP@0.5 scores. We performed comparative analyses with YOLOv5s, YOLOv5m, YOLOv5n, YOLOv8s, and YOLOv8n using our model and consistently found higher mAP@0.5 scores with our improved model. Furthermore, we evaluated the efficiency of our enhanced model in terms of detection accuracy compared to YOLOv8s, confirming the superiority of our approach.

### 4.3. Ablation Study

#### 4.3.1. Comparison of the Effectiveness of the LarK Block at Different Positions

Table 7 compares the impact of using the LarK block to replace different positions of the C2f in the backbone on the accuracy, the number of parameters, and the computational complexity of the model on the URPC2019 dataset, for various categories of marine life. Among them, the model with the middle two C2fs in the backbone replaced by the LarK block performed the best, achieving an mAP@0.5 of 75.5%, with the smallest number of parameters, similar to the model in which only the last C2f was modified, and with FLOPs at a medium level. In contrast, the model in which only the last C2f was modified had the smallest number of parameters and the lowest computational complexity but experienced a decrease in accuracy compared to the original YOLOv8n. The accuracy of other models with different modification positions was also lower than that of the original YOLOv8n. Therefore, in the subsequent research, we adopted the model that replaced the middle two C2fs with the LarK block, as it ensured higher accuracy while improving the speed of the object detection model, with a smaller modification to the network.

#### 4.3.2. Comparison of the Effectiveness of the C2fSTR at Different Positions

Table 8 compares the impact of using the C2fSTR to replace different positions of the C2f in the backbone on the accuracy, the number of parameters, and the computational complexity of the model on the URPC2019 dataset, for various categories of marine life. Among them, the model with the last C2f in the backbone replaced by C2fSTR performed the best, achieving an mAP@0.5 of 75.2%, with the smallest computational load and the fastest speed. In contrast, the model that replaced all C2fs had a decrease in accuracy, with an mAP@0.5 of only 73.8%. Other models with different modification positions, although all having an mAP@0.5 higher than YOLOv8n, did not perform as well as the model in which only the last C2f was modified regarding the computational load and speed. Therefore, in our subsequent research, we adopted the model that replaced the last C2f with the C2fSTR, as it ensured the highest accuracy while also achieving the best computational efficiency and speed.

#### 4.3.3. Comparison of the Effectiveness of the Fusion Block at Different Positions

Table 9 shows the impact of using the fusion block to replace different positions of the C2f in the neck on the accuracy, the number of parameters, and the computational complexity of the model on the URPC2019 dataset, for various categories of marine life. Among them, the model with all C2fs in the neck replaced by the fusion block performed the best, achieving an mAP@0.5 of 74.7%; although its number of parameters and computational complexity were not the lowest, its accuracy was the highest. In comparison, the models in which we modified the last three C2fs and the middle two C2fs had smaller parameter counts and lower computational complexity but mAP@0.5 values of only 74.1% and 73.5%, respectively, which were 0.3% and 0.9% lower than those of YOLOv8n. Modifications at other positions also failed to improve the model’s accuracy compared to the modification of all C2fs. Therefore, in our subsequent research, we adopted the model that replaced all C2fs with the fusion block, as it achieved higher target detection accuracy.

#### 4.3.4. Analysis of the Effectiveness of Other Modules

In this section, we took the original YOLOv8 as the base and gradually added or removed the components included in our model to explore the contribution of each component to the overall performance of the system model, thereby demonstrating their effectiveness in improving YOLOv8. We conducted multiple ablation experiments, and, by analyzing Table 10, we can see that different combinations of modules had varying effects on the performance of the YOLOv8 model.

In the process of optimizing the YOLOv8 model, we first added five modules individually, and the mAP@0.5 values obtained were all improved compared to the original YOLOv8, with the improvement effects ranked from largest to smallest as follows: LarK block, SPPFCSPC_EMA, C2fSTR, fusion block, and MPDIoU. It can be seen that the use of the LarK block module alone resulted in the highest increase in the mAP@0.5, which was 1.1%. This indicates that all five modules had a positive impact in optimizing the detection accuracy of YOLOv8.

When these modules are used in combination, the mAP@0.5 also increases, and the increase in mAP@0.5 is generally greater compared to when each module is used individually. The best combination is when the LarK block, SPPFCSPC_EMA, C2fSTR, fusion block, and MPDIoU are used simultaneously, achieving the highest mAP@0.5 of 78.4%, which is an increase of 4.0% compared to the original YOLOv8. In summary, based on the experimental results, the simultaneous use of the LarK block, SPPFCSPC_EMA, C2fSTR, fusion block, and MPDIoU can produce the best performance improvement. These results provide guidance for the design and configuration of optimized object detection systems.

### 4.4. Analysis of Results

**Figure 16:**
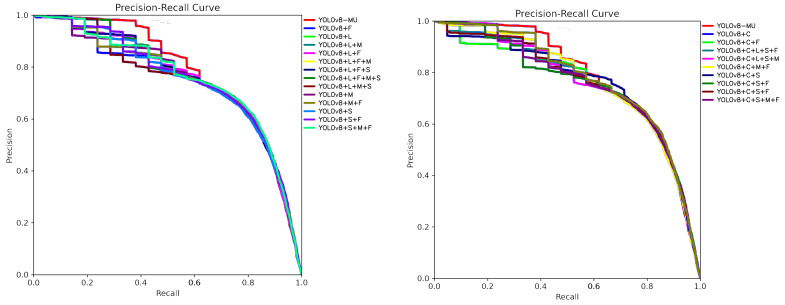
Summary of PR curves of each model in the ablation experiment. ‘+L’ corresponds to the LarK block; ‘+C’ corresponds to ‘C2fSTR’; ‘+S’ corresponds to SPPFCSPC_EMA; ‘+F’ corresponds to the fusion block; ‘+M’ corresponds to MPDIOU.

To further prove the effectiveness of each module of YOLOv8-MU, we summarized and compared the experimental results of each ablation experiment on the dataset URPC2019. We took all class mAP@0.5 PR curves from each precision–recall (PR) curve and summarized them in the same coordinate system, as shown in Figure 16. From the figures, we can see that after our model is added to the original YOLOv8 model, the PR curve as a whole moves closer to the upper-right corner, which indicates that, after the addition of each module, the performance of the improved YOLOv8 changes in a positive direction, which further proves the effectiveness of each improved module.

## 5. Conclusions and Future Work

In this study, we have successfully developed and validated an advanced underwater organism detection framework named YOLOv8-MU, which significantly improves the detection accuracy. By replacing the original backbone network structure with the LarK block proposed in UniRepLKNet, we obtain a larger receptive field without increasing the model’s depth. Integrating the Swin Transformer module into the C2f module further enhances the model’s capability to learn and generalize to various underwater biological features. Combining the multi-scale attention module EMA with SPPFCSPC significantly improves the detection accuracy and robustness for multi-scale targets. Introducing a fusion block into the neck network enhances the model’s feature extraction and integration capabilities across different scales. By utilizing the MPDIoU loss function, which is optimized based on the vertex distance, we effectively address target localization and boundary precision issues, thereby enhancing the detection accuracy. Validation on the URPC2019 and URPC2020 datasets demonstrates that the YOLOv8-MU model achieves mAP@0.5 scores of 78.4% and 80.9%, respectively, representing improvements of 4.0% and 0.9% over the YOLOv8n model. These achievements not only validate the effectiveness of our proposed method but also provide new research directions and practical foundations for the development of target detection technology in complex environments. Additionally, the evaluation of the refined URPC2019 dataset demonstrates leading performance (SOTA), with an mAP@0.5 of 88.1%, further confirming the superiority of the model on this dataset. These results highlight the extensive applicability and generalization capabilities of the model across various underwater datasets.

## Figures and Tables

**Table 1:** Experimental environment configuration.

Parameter	Setup
Ubuntu	20.04
PyTorch	1.11.0
Python3	3.8
CUDA	11.3
CPU	12 vCPU Intel(R) Xeon(R) Platinum 8255C CPU @ 2.50 GHz (Intel, Santa Clara, CA, USA)
GPU	RTX 3090 (24 GB) × 1
RAM	43 GB

**Table 2:** Settings of some hyperparameters during training.

Parameter	Setup
Epoch	100
Batch Size	16
NMS IoU	0.7
Image Size	640 × 640
Initial Learning Rate	$1\times 10^{-2}$
Final Learning Rate	$1\times 10^{-2}$
Momentum	0.937
Weight Decay	0.0005

**Table 3:** Performance comparison of the YOLOv8-MU model and other models on the URPC2019 dataset.

Model	AP (%)					mAP@0.5 (%)	Para (M)	FLOPs (G)
	Echinus	Starfish	Holothurian	Scallop	Waterweeds			
Boosting R-CNN [37]	89.2	86.7	72.2	76.4	26.6	70.2	45.9	77.6
Improved YOLOv5 [73]	92.3	88.4	77.3	85.0	35.3	75.7	139.9	28.0
YOLOv3	89.6	86.8	73.6	82.6	57.8	78.1	61.5	155.3
YOLOv5s	92.0	88.1	75.2	84.5	24.2	72.8	20.9	47.9
YOLOv5m	91.9	86.3	58.4	71.8	17.6	62.5	1.8	4.2
YOLOv5n	92.4	89.3	74.7	83.8	28.4	73.7	7.0	16.0
YOLOv7	92.6	90.0	78.5	85.6	39.6	77.3	37.2	105.2
YOLOv8s	91.3	89.0	75.2	84.9	32.1	74.5	11.1	28.4
YOLOv8m	90.9	89.5	76.9	85.7	28.1	74.2	25.9	79.1
YOLOv8l	90.9	90.4	77.1	84.8	27.0	74.0	43.6	165.4
YOLOv8n	91.7	89.2	76.1	82.8	32.3	74.4	3.0	8.2
YOLOv8-MU	91.9	89.3	75.8	83.5	51.5	78.4	5.7	28.7

**Table 4:** The mAP@0.5 comparison of four class objects on the URPC2019 dataset.

Model	Num	Train	Valid	Test	mAP@0.5 (%)
Faster R-CNN (VGG16)	4757	3805	0	952	66.5
SSD (VGG16) [48]	4757	3805	0	952	70.2
FCOS (ResNet50) [74]	4757	3805	0	952	72.3
Faster R-CNN (ResNet50)	4757	3805	0	952	73.2
FreeAnchor (ResNet50) [75]	4757	3805	0	952	74.8
EfficientDet [76]	4757	3805	0	952	74.8
CenterNet (ResNet50) [77]	4757	3805	0	952	76.1
YOLOv8n	4707	3765	0	942	86.1
DDA+YOLOv7-ACmix [78]	4707	3765	0	942	87.2
Ours	4707	3765	0	942	88.1

**Table 5:** Performance comparison of the YOLOv8-MU model and other models on the URPC2020 dataset.

Model	AP (%)				mAP@0.5 (%)
	Echinus	Starfish	Holothurian	Scallop	
Improved YOLOv5	69.7	89.0	76.7	84.2	79.9
SA-SPPN [79]	69.7	88.5	74.7	83.0	79.0
YOLOv8s	72.0	89.4	78.7	83.9	81.0
Ours	73.4	89.9	79.4	84.5	81.7
YOLOv8n	77.1	89.8	75.7	83.6	80.0
Ourn	71.0	89.4	78.5	84.5	80.9

**Table 6:** Performance comparison of the YOLOv8-MU model and other models on the Aquarium dataset.

Model	AP (%)							mAP@0.5 (%)
	Fish	Jellyfish	Penguin	Puffin	Shark	Starfish	Stingray	
YOLOv5s	75.3	90.2	67.1	53.5	68.9	72.5	81.7	72.8
YOLOv5m	77.3	90.0	64.0	58.2	67.2	71.3	76.1	72
YOLOv5n	78.1	93.1	70.1	61.3	74.2	69.8	75.5	74.6
YOLOv8s	69.2	88.5	61.3	47.6	58.2	68.4	77.6	67.2
YOLOv8n	80.1	92.0	65.7	50.7	68.5	75.4	81.4	73.4
Ourn	78.7	91.7	69.0	58.6	73.0	77.8	79.9	75.5

**Table 7:** Parameter comparison when replacing the C2f with the LarK block at different positions in the backbone.

Location of LarK Block	AP (%)					mAP@0.5 (%)	Para (M)	FLOPs (G)
	Echinus	Starfish	Holothurian	Scallop	Waterweeds			
All	91.5	88.0	73.5	82.1	35.8	74.2	3.4	9.7
The last three	91.8	88.8	73.0	82.6	30.1	73.3	3.4	9.3
The last two	90.7	88.8	75.2	82.8	29.4	73.4	3.4	8.7
The last one	91.7	89.5	75.6	83.6	28.9	73.9	3.2	8.2
The middle two	92.2	89.4	76.4	84.6	34.7	75.5	3.2	9.2

**Table 8:** Parameter comparison when replacing the C2f with the C2fSTR at different positions in the backbone.

Location of C2fSTR	AP (%)					mAP@0.5 (%)	Para (M)	FLOPs (G)
	Echinus	Starfish	Holothurian	Scallop	Waterweeds			
All	90.5	89.1	73.6	82.1	33.8	73.8	3.0	30.9
The last three	90.9	88.6	75.5	82.3	36.2	74.7	3.0	29.9
The last two	90.4	88.9	75.4	82.8	35.0	74.5	3.0	27.8
The last one	91.4	88.9	75.7	82.4	37.6	75.2	2.9	18.1
The middle two	91.6	89.0	73.2	81.9	38.4	74.8	3.1	20.0

**Table 9:** Parameter comparison when replacing the C2f with the fusion block at different positions in the neck.

Location of Fusion Block	AP (%)					mAP@0.5 (%)	Para (M)	FLOPs (G)
	Echinus	Starfish	Holothurian	Scallop	Waterweeds			
All	91.5	89.7	75.7	84.0	32.6	74.7	3.95	16.5
The last three	92.2	89.6	75.6	83.8	29.1	74.1	3.8	15.8
The last two	91.8	89.1	75.7	83.1	32.9	74.5	2.9	8.4
The last one	92.0	88.8	75.3	83.3	33.5	74.6	2.7	7.8
The middle two	92.1	89.9	75.3	83.2	26.9	73.5	2.9	8.4

**Table 10:** Demonstration of the effectiveness of each module in YOLOv8-MU. ‘√’ indicates that we use this module.

Module					mAP@0.5 (%)
LarK Block	C2fSTR	SPPFCSPC_EMA	Fusion Block	MPDIOU	
√					75.5
	√				75.2
		√			75.3
			√		74.7
				√	74.6
√			√		75.6
√				√	75.7
	√	√			75.6
	√		√		75.6
	√			√	75.7
		√	√		75.4
		√		√	75.6
			√	√	75.5
	√	√	√		75.8
√		√		√	76.3
√		√	√		75.8
	√	√		√	75.8
		√	√	√	75.9
	√		√	√	76.0
√	√	√	√		76.0
√	√	√		√	76.5
	√	√	√	√	77.6
√		√	√	√	76.4
√	√	√	√	√	78.4

## Data Availability

The datasets generated during and/or analyzed during the current study are available from the corresponding authors upon reasonable request.
